# Nomogram for predicting 1-, 5-, and 10-year survival in hemodialysis (HD) patients: a single center retrospective study

**DOI:** 10.1080/0886022X.2021.1997762

**Published:** 2021-11-15

**Authors:** Han Ouyang, Qiuhong Shi, Jing Zhu, Huaying Shen, Shan Jiang, Kai Song

**Affiliations:** a Department of Nephrology, The Second Affiliated Hospital of Soochow University, Suzhou, China; b Department of Cardiology, The Second Affiliated Hospital of Soochow University, Suzhou, China

**Keywords:** Hemodialysis, nomogram, prediction model, survival, all-cause mortality

## Abstract

**Objectives:**

Risk of death is high for hemodialysis (HD) patients but it varies considerably among individuals. There is few clinical tool to predict long-term survival rates for HD patients yet. The aim of this study was to develop and validate a easy-to-use nomogram for prediction of 1-, 5-, and 10-year survival among HD patients.

**Methods:**

This study retrospectively enrolled 643 adult HD patients who was randomly assigned to two cohorts: the training cohort (*n* = 438) and validation cohort (*n* = 205), univariate survival analyses were performed using Kaplan–Meier’s curve with log-rank test and multivariate Cox regression analyses were performed to identify predictive factors, and a easy-to-use nomogram was established. The performance was assessed using the area under the curve (AUC), calibration plots, and decision curve analysis.

**Results:**

The score included seven commonly available predictors: age, diabetes, use of arteriovenous fistula (AVF), history of emergency temporary dialysis catheter placement, cardiovascular disease (CVD), hemoglobin (Hgl), and no caregiver. The score revealed good discrimination in the training and validation cohort (AUC 0.779 and 0.758, respectively) and the calibration plots showed well calibration, indicating suitable performance of the nomogram model. Decision curve analysis showed that the nomogram added more net benefit compared with the treat-all strategy or treat-none strategy with a threshold probability of 10% or greater.

**Conclusions:**

This easy-to-use nomogram can accurately predict 1-, 5-, and 10-year survival for HD patients, which could be used in clinical decision-making and clinical care.

****Abbreviations**:**

## Introduction

The incidence of chronic kidney disease (CKD) is increasing year by year on a global scale and the all-cause mortality in dialysis patients is 6.5–7.9 times higher than in the general population [1], about 1% of CKD progresses to end-stage renal disease (ESRD) and requires renal replacement therapy and about 90% of ESRD patients in China received hemodialysis (HD) treatment [2,3]. In recent years, with the development of HD technology and the improvement of management, the survival rates of HD patients have been continuously improved; however, there were still many patients that die from HD-related complications. For example, cardiovascular complications, infections, and sudden death are the leading causes of death in patients undergoing HD [4]. Although we know that risk factors for death in HD patients are associated with traditional risk factors such as age, sex, smoking, hypertension, diabetes, hyperlipidemia, and nontraditional risk factors such as anemia, inflammation and oxidative stress, uremia toxins, and dysregulation of calcium and phosphorus metabolism [5], how to accurately screen out different death risk factors of different individuals among these death factors? How to predict the death risk of patients at the beginning of dialysis? How to establish an effective clinical prediction model and implement intervention for clinicians at an early stage? These problems are still not well solved.

As a mathematical model to predict the probability of end-point events, risk prediction model has been widely used in the medical field, for example, the EuroSCORE II model for predicting the risk of heart surgery and the Charlson comorbidity index (CCI) for predicting survival of cancer patients [6,7]. In previous studies, Mauri et al. [8] used Logistic regression to determine the risk factors for death after 1 year of dialysis, and then validated the prognostic model and quantified the risk of death for each HD patient. Floege et al. [9] used Cox regression to establish a risk prediction model and established a prediction model of mortality risk scores of HD patients after 1 year and 2 years of dialysis in a region of Europe, meanwhile, Wagner et al. [10] used Cox regression risk model to predict the death risk of British HD patients after 3 years of dialysis. Although these clinical prediction models can effectively predict the death risk of HD patients to a certain extent, they have the disadvantage that they are all short-term predictions and lack the risk prediction data for long-term survival of HD patients. Meanwhile, these prediction methods are not simple and intuitive enough for clinicians. In this study, we sought to develop and validate a easy-to-use nomogram for prediction of near-term, intermediate-term, and long-term mortality from any cause.

### Patients

**Figure 1.**
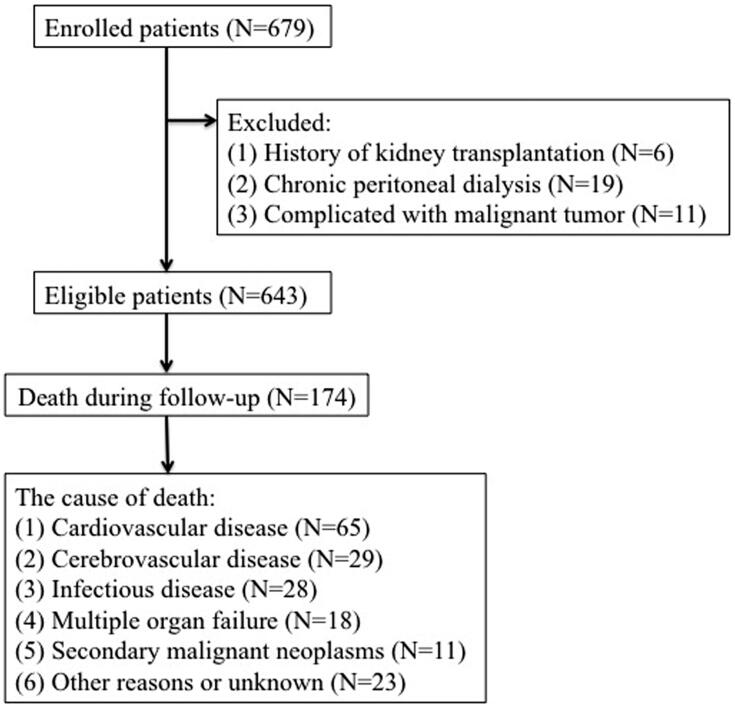
Enrollment and outcomes of the cohort.

This study enrolled 679 adult HD patients from the Second Affiliated Hospital of Soochow University in China from 31 January 2009 to 31 December 2013. The exclusion criteria were as follows: (1) history of kidney transplantation; (2) chronic peritoneal dialysis (PD); (3) complicated with malignant tumor; after eliminations, 643 subjects were enrolled in our analysis (Figure 1). The final cohort was randomly divided in a training cohort (*n* = 438) and a validation cohort (*n* = 205). The study protocol was approved by the Clinical Research Ethics Committee of The Second Affiliated Hospital of Soochow University and is registered in the Chinese Clinical Trial Registry (no. ChiCTR1900024999).

### Clinical and laboratory parameters

We recorded the following laboratory parameters: creatinine (Cr), hemoglobin (Hgl), albumin (Alb), blood urea nitrogen (BUN), serum uric acid (UA), calcium (Ca), phosphorus (P), potassium (K), low-density lipoprotein cholesterol (LDL), total triglycerides (TGs), total cholesterol (TC), parathyroid hormone (PTH), high-sensitivity C-reactive protein (Hs-CRP), residual kidney function (RKF), and total *Kt*/*V* (*Kt*/*V* is the fractional urea clearance, defined as urea clearance (*K*) multiplied by the dialysis session length (*t*) divided by the urea distribution volume *V*). The *Kt*/*V* results were obtained by the special HD formula of *Kt*/*V*. RKF was estimated from mean values of creatinine clearance and urea clearance.

### Candidate variables

Demographic variables were included in candidate variables, such as age, smoking, and gender; blood pressure (systolic blood pressure and diastolic blood pressure), height and dialysis dry weight were included as physical examination variables; concurrent disease, including diabetes, hypertension, and cardiovascular disease (CVD). These data were obtained on admission in the diagnostic laboratory of the Second Affiliated Hospital of Soochow University. All data were obtained within 1 months after the patient’s regular HD reached dry body mass. Body mass index (BMI) was calculated according to the height and weight. Diabetes was defined by use of hypoglycemic medications (and/or use of insulin) and/or history of clinical diagnosis. Hypertension was based on at least two separate blood pressure measurements ≥130/80 mmHg or prescription of antihypertensive drugs. History of angina pectoris, myocardial infarction, heart failure, coronary artery bypass, angioplasty, arrhythmia, or stroke taken from clinical records were considered as CVD.

### Follow-up procedures and outcome

Individuals belonging to the training and validation cohort were followed for a median 7.9 years (5.5–10.4 years), for mortality from any cause. The outcome of interest was all-cause mortality which was defined as death due to CVD; cerebrovascular disease; infectious disease; multiple organ failure; secondary malignant neoplasms; other reasons or unknown, as shown in Figure 1. If death happened outside a hospital, death classifications required independent audits by two experts in our dialysis center after a comprehensive consideration of descriptions of caregivers and the patient’s medical records. All patients were followed up until death, transfer to PD treatment, undergoing a renal transplant, or transfer to another dialysis center on 31 December 2013.

### Statistical analysis

The final cohort was randomly divided into a training cohort (*n* = 438) and a validation cohort (*n* = 205). Continuous variables were presented as mean ± SD or median (interquartile range) and compared using an ANOVA or the Kruskal–Wallis test as appropriate. Categorical variables were given as proportions and compared using a *χ*^2^ test. The 24-h urine output, age and Hgl were transformed into categorical variables. The remainder of the variables were evaluated as linear predictors. The Cox regression model was finally used to determine variable selection and constructed a model for predicting all-cause mortality in the training cohort. The hazard ratio (HR) and the 95% confidence interval (95% CI) were calculated as well. *p*<.05 was considered statistically significant for tests.

Univariate survival analyses of the grouping variables were performed using Kaplan–Meier’s curve with log-rank test. Multivariable analysis was performed using the Cox regression models to develop a nomogram. The predictive performance of the nomograms was evaluated by the area under the curve (AUC). Calibration was performed using bootstrapping with 1000 research resamples and assessed utilizing calibration plots, which measured the relationship between predicted probabilities and observed proportions. Decision curve analysis was conducted to determine the clinical usefulness of the survival nomogram by quantifying the net benefits at different threshold probabilities in the cohort. By using recursive partitioning tree analysis to generate the optimum cut of point, patients were categorized as ‘low’ or ‘high’ risk group, and the Kaplan–Meier curves were plotted for these two risk groups. Statistical analyses were performed using the SPSS software (version 23.0, SPSS Inc., Chicago, IL) and R software (version 3.6.2, Vienna, Austria).

## Results

### Baseline characteristics

The baseline characteristics of the two groups are summarized in Table 1. Patients in the training and validation cohorts were similar in demographic characteristics, comorbidities, laboratory data, use of medicine and outcomes (Table 1). The pathogenesis of ESRD included glomerulonephritis (299/643), diabetic nephropathy (168/643), polycystic kidney disease (18/643), urinary tract infection (9/643), gouty nephropathy (6/643), urine tract obstruction (4/643), IgA nephropathy (69/643), tumor-related nephropathy (11/643), renal tuberculosis (8/643), congenital renal atrophy (4/643), and with unidentified causes in 47 patients. One hundred and seventy-four patients died by the end of the follow-up and 65 (37.36%) deaths were attributed to CVD, the detailed causes of death are shown in Figure 1. The clinical features and laboratory indicators of HD patients with mortality end points were compared with surviving patients (Table 2).

**Table 1.** Baseline characteristics of the study populations and subpopulations.

Characteristic	Training cohort (*n* = 438)	Validation cohort (*n* = 205)	*p* Value
Age at diagnosis (years), mean ± SD	57.68 ± 15.81	57.19 ± 16.53	.423
Male	213 (48.63)	98 (47.80)	.411
Body mass index (BMI) (kg/m^2^)	21.79 ± 3.21	21.97 ± 3.03	.197
Smokers, *n* (%)	53 (12.10)	23 (11.22)	.471
Diabetes, *n* (%)	136 (31.05)	66 (32.20)	.283
Hypertension, *n* (%)	401(91.55)	188 (91.71)	.189
CVD, *n* (%)	158 (36.07)	73 (35.61)	.653
Systolic blood pressure (mmHg)	142.82 ± 21.35	143.51 ± 20.05	.592
Diastolic blood pressure (mmHg)	85.34 ± 32.42	82.15 ± 12.83	.356
Serum creatinine (59–104 µmol/L)	812.32 ± 353.73	794.06 ± 355.82	.070
Serum uric acid (89–420 µmol/L)	446.82 ± 311.62	434.01 ± 156.46	.783
Blood urea nitrogen (2.8–7.1 mmol/L)	24.64 ± 12.29	23.96 ± 11.44	.502
Hemoglobin (120–160 g/L)	89.79 ± 22.55	88.05 ± 22.81	.486
White blood cell (3.5–9.5 × 10^9^/L)	6.38 ± 3.13	6.69 ± 3.53	.526
Serum albumin (35–55 g/L)	33.83 ± 6.86	32.99 ± 6.59	.829
Serum calcium (2–2.7 mmol/L)	2.08 ± 0.52	2.07 ± 0.28	.650
Serum phosphorus (0.81–1.55 mmol/L)	1.86 ± 0.59	1.82 ± 0.62	.981
Serum potassium (3.5–5.5 mmol/L)	4.31 ± 0.82	4.36 ± 0.91	.443
Triglycerides (0.3–1.7 mmol/L)	1.53 ± 1.19	1.41 ± 0.76	.342
Total cholesterol (0–5.69 mmol/L)	4.19 ± 1.46	4.35 ± 1.26	.553
Low density lipoprotein (0.2–3.1 mmol/L)	2.41 ± 1.23	2.49 ± 0.96	.432
Hs-CRP (0–10 mg/L)	21.27 ± 34.81	21.35 ± 34.06	.990
PTH (pg/mL) (12–88 pg/mL)	337.54 ± 310.06	333.12 ± 398.88	.258
24 hours urine output < 400 mL	175 (39.96)	88 (42.93)	.184
*Kt*/*V*	1.21 ± 0.22	1.29 ± 0.27	.447
RKF (mL/min/1.73 m^2^)	6.69 ± 4.51	6.52 ± 3.58	.313
ACEi/ARB, *n* (%)	146 (33.33)	66 (32.20)	.237
CCB, *n* (%)	298 (68.04)	134 (65.37)	.100
Dialysis access			
AVF, *n* (%)	329 (75.11)	139 (67.80)	.090
Semi-permanent dialysis catheter, *n* (%)	157 (24.42)	63 (30.73)	.110
Emergency temporary dialysis catheter placement, *n* (%)	240 (54.79)	122 (59.51)	.239
No caregiver	49 (11.19)	27 (13.17)	.468
Death, *n* (%)	116 (26.48)	58 (28.29)	.607

BMI: body mass index; CVD: cardiovascular disease; HB: hemoglobin; ACEi: angiotensin-converting enzyme inhibitor; ARB: angiotensin receptor blocker; CCB: calcium channel blocker; Hs-CRP: high-sensitivity C-reactive protein; PTH: parathyroid hormone; RKF: residual kidney function; AVF: arteriovenous fistula.

**Table 2.** The comparison of clinical characteristics in the dead and the living patients.

Characteristic	Death (*n* = 174)	Survival (*n* = 469)	*p* Value
Age at diagnosis (years), mean ± SD	65.03 ± 13.75	54.55 ± 16.26	<.001
Male	81 (46.55)	228 (48.61)	.073
BMI (kg/m^2^)	21.69 ± 2.71	21.87 ± 3.13	.501
Smokers, *n* (%)	19 (10.92)	53 (11.30)	.892
Diabetes, *n* (%)	80 (45.98)	129 (27.51)	<.001
Hypertension, *n* (%)	163 (93.68)	440 (93.82)	.949
CVD, *n* (%)	93 (53.45)	144 (30.70)	<.001
Systolic blood pressure (mmHg)	147.25 ± 21.67	145.28 ± 20.25	.282
Diastolic blood pressure (mmHg)	80.98 ± 13.41	85.34 ± 35.54	.114
Serum creatinine (59–104 µmol/L)	806.42 ± 316.84	884.38 ± 389.74	.047
Serum uric acid (89–420 µmol/L)	445.47 ± 153.74	449.06 ± 300.58	.880
Blood urea nitrogen (2.8–7.1 mmol/L)	23.57 ± 11.47	24.57 ± 11.09	.314
Hemoglobin (120–160 g/L)	84.87 ± 20.63	88.99 ± 22.73	.030
White blood cell (3.5–9.5 × 10^9^/L)	7.298 ± 3.9907	6.67 ± 2.40	.042
Serum albumin (35–55 g/L)	32.98 ± 7.09	33.15 ± 6.57	.137
Serum calcium (2–2.7 mmol/L)	2.03 ± 0.27	2.09 ± 0.54	.183
Serum phosphorus (0.81–1.55 mmol/L)	1.71 ± 0.54	1.89 ± 0.61	.040
Serum potassium (3.5–5.5 mmol/L)	4.38 ± 0.84	4.36 ± 0.84	.739
Triglycerides (0.3–1.7 mmol/L)	1.49 ± 1.22	1.61 ± 1.26	.295
Total cholesterol (0–5.69 mmol/L)	4.36 ± 1.43	4.31 ± 1.32	.678
Low density lipoprotein (0.2–3.1 mmol/L)	2.53 ± 1.23	2.46 ± 1.01	.435
Hs-CRP (0–10 mg/L)	23.65 ± 45.45	21.68 ± 29.85	.042
PTH (pg/mL) (12–88 pg/mL)	369.27 ± 277.46	354.57 ± 366.52	.253
24 hours urine output < 400 mL	78 (44.83)	174 (37.10)	.075
*Kt*/*V*	1.13 ± 0.24	1.19 ± 0.26	.157
RKF (mL/min/1.73 m^2^)	7.39 ± 4.77	7.26 ± 4.21	.366
ACEi/ARB, *n* (%)	70 (40.23)	158 (33.69)	.123
CCB, *n* (%)	122 (70.11)	336 (71.64)	.249
Dialysis access			
AVF, *n* (%)	81 (46.55)	384 (81.88)	<.001
Semi-permanent dialysis catheter, *n* (%)	93 (53.45)	85 (18.12)	<.001
Emergency temporary dialysis catheter placement, *n* (%)	113 (64.94)	248 (52.88)	.006
No caregiver	30 (17.24)	46 (9.81)	.009
Death, *n* (%)	174 (100)	0 (0)	<.001

BMI: body mass index; CVD: cardiovascular disease; HB: hemoglobin; ACEi: angiotensin-converting enzyme inhibitor; ARB: angiotensin receptor blocker; CCB: calcium channel blocker; Hs-CRP: high-sensitivity C-reactive protein; PTH: parathyroid hormone; RKF: residual kidney function; AVF: arteriovenous fistula.

### The results of the selection of variables

**Figure 2.**
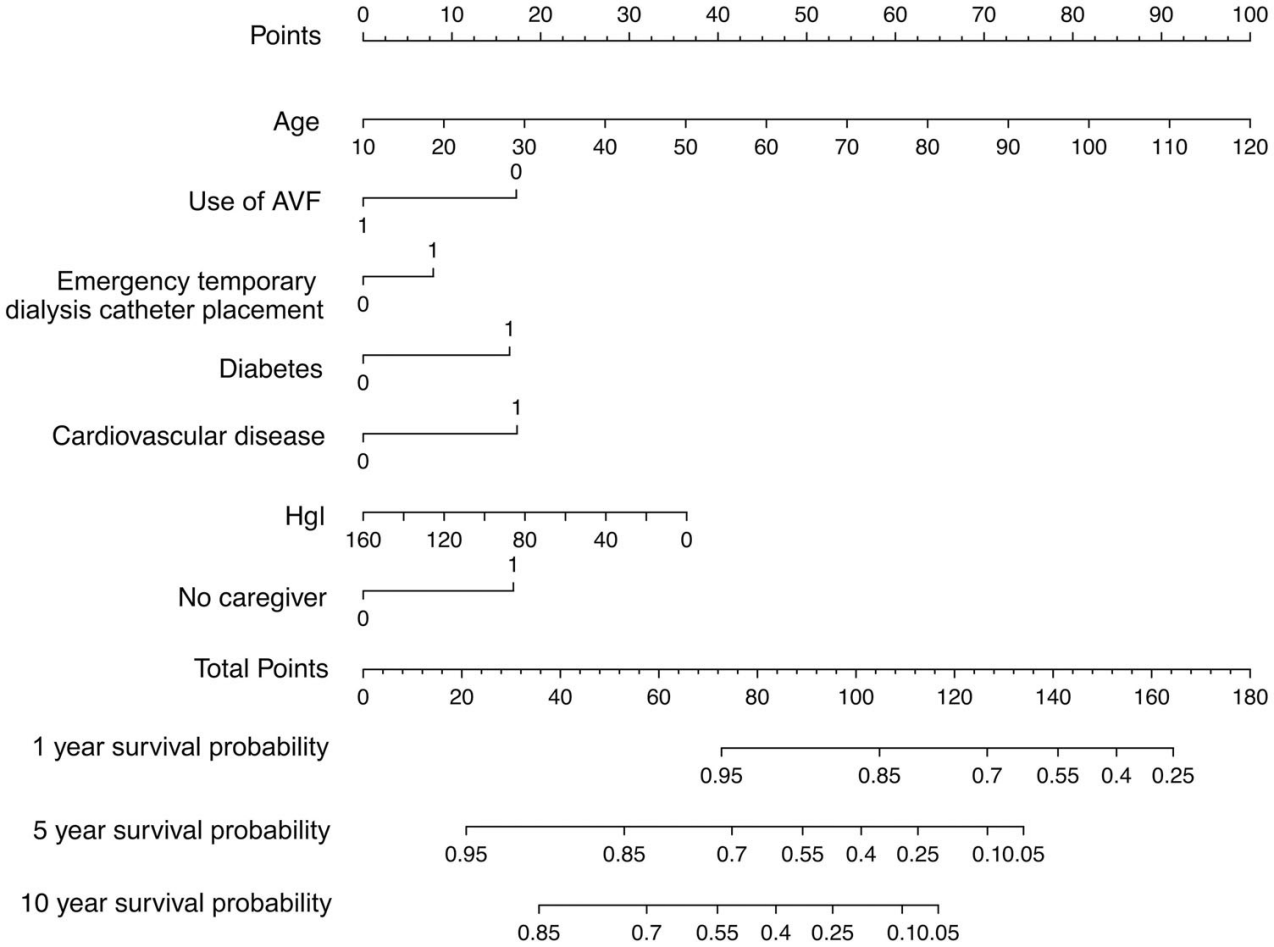
Nomogram to predict risk of all-cause mortality in HD patients.

In training cohort, univariable analysis found 12 candidate predictors that were closely associated with the all-cause mortality (Table 1), including ‘age’, ‘diabetes’, ‘CVD’, ‘serum Cr’, ‘Hgl’, ‘Hs-CRP’, ‘white blood cell’, ‘serum P’, ‘no caregiver’, ‘use of AVF’, ‘use of polyester cuffed semi-permanent dialysis catheter’ and ‘history of emergency temporary dialysis catheter placement’. After multivariable Cox regressive analysis, seven predictors were left for inclusion in the final multivariable model (Table 3): ‘age’, ‘diabetes’, ‘CVD’, ‘Hgl’, ‘no caregiver’, ‘use of AVF’ (because of HD patients either use arteriovenous fistula (AVF) or semi-permanent dialysis catheter, so, the two variables of ‘use of AVF’ and ‘use of polyester cuffed semi-permanent dialysis catheter’ have the same predictive value, and ‘use of AVF’ was selected as the predictive variable in this study), and ‘history of emergency temporary dialysis catheter placement’. Based on these seven predictors, we used a nomogram to develop a score for the prediction of survival (Figure 2).

**Table 3.** Multivariable hazard ratios for the relationship between prognostic risk factors and 10-, 5-, and 1-year all-cause mortality.

Categories	10-year follow-up	5-year follow-up	1-year follow-up
	HR (95% CI), *p*	HR (95% CI), *p*	HR (95% CI), *p*
Training cohort			
Age	1.035 (1.023, 1.047), <.001	1.030 (1.018, 1.042), <.001	1.015 (0.995, 1.306), .132
Diabetes	0.580 (0.428, 0.787), <.001	0.476 (0.347, 0.654), <.001	0.566 (0.329, 0.973), .39
Use of AVF	1.825 (1.318, 2.527), <.001	1.936 (1.381, 2.715), <.001	3.744 (2.022, 7.046), <.001
No caregiver	0.535 (0.359, 0.799), .002	0.649 (0.422, 0.998), .049	0.717 (0.335, 1.535), .391
Emergency temporary dialysis catheter placement	0.703 (0.505, 0.980), .038	0.795 (0.569, 1.110), .178	0.439 (0.223, 0.865), .017
CVD	0.538 (0.392, 0.738), <.001	0.505 (0.361, 0.707), <.001	0.692 (0.390, 1.227), .208
Hgl	0.992 (0.985, 0.999), .031	0.990 (0.983, 0.998), .015	0.984 (0.970, 0.998), .028
Validation cohort			
Age	1.033 (1.021, 1.045), <.001	1.032 (1.020, 1.045), <.001	1.016 (0.996, 1.036), .126
Diabetes	0.555 (0.409, 0.752), <.001	0.536 (0.391, 0.735), <.001	0.674 (0.391, 1.160), .155
Use of AVF	1.850 (1.340, 2.553), <.001	1.952 (1.388, 2.745), <.001	3.915 (2.082, 7.360), <.001
No caregiver	0.548 (0.368, 0.815), .03	0.645 (0.419, 0.993), .047	0.714 (0.332, 1.532), .387
Emergency temporary dialysis catheter placement	0.755 (0.548, 1.039), .084	0.714 (0.505, 1.010), .057	0.403 (0.198, 0.820), .012
CVD	0.602 (0.440, 0.823), .001	0.526 (0.377, 0.374), <.001	0.821 (0.468, 1.438), .490
HB	0.813 (0.632, 1.044), .15	0.820 (0.630, 1.069), .143	0.684 (0.441, 1.059), .048

Hgl: hemoglobin; AVF: arteriovenous fistula; HR: hazard ratios; CVD: cardiovascular disease.

### Nomogram for predicting survival

Multivariable Cox regression and HRs were calculated for the prognostic factors used to establish the nomogram (Table 3). In the training cohort, increasing age, CVD, severe anemia, diabetes, no caregiver, use of AVF, and history of emergency temporary dialysis catheter placement were associated with survival from all causes across 10 years of study follow-up, these relationships were similar in the validation cohort. The relationship between the prognostic factors and risk of all-cause death was roughly similar when reevaluating the association at 1- and 5-year study follow-up. The linear predictors from the Cox regression model were used to develop the nomogram to predict survival in HD patients (Figure 2).

### Validation nomogram

**Figure 3.**
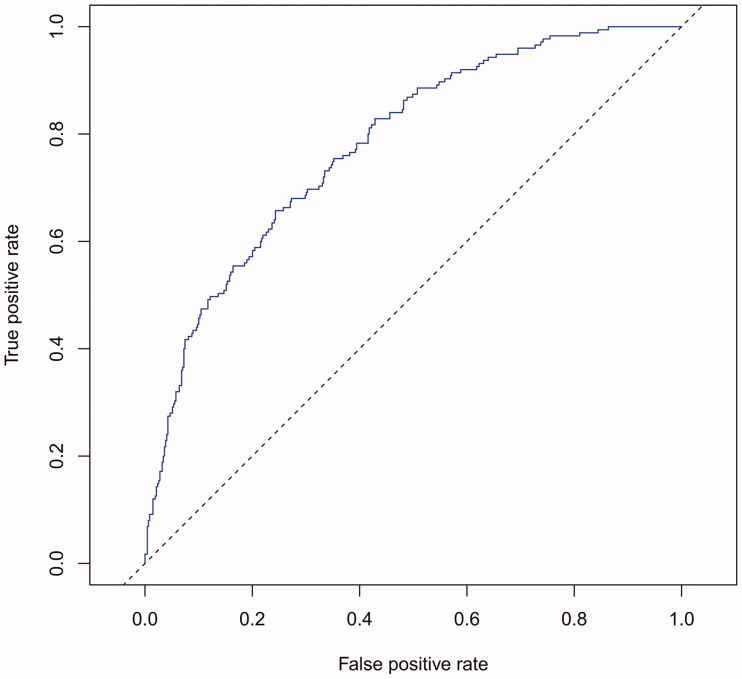
AUC of training cohort was 0.779.

**Figure 4.**
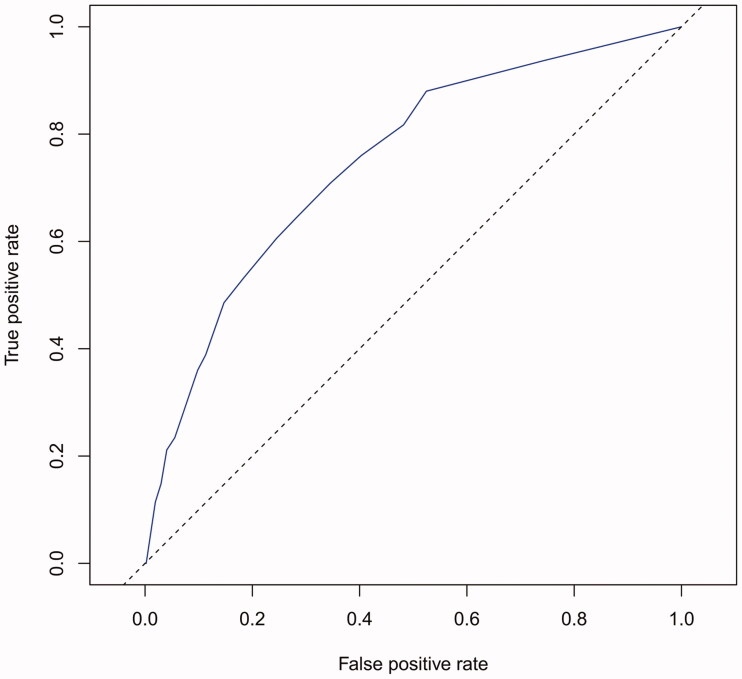
AUC of validation cohort was 0.758.

**Figure 5.**
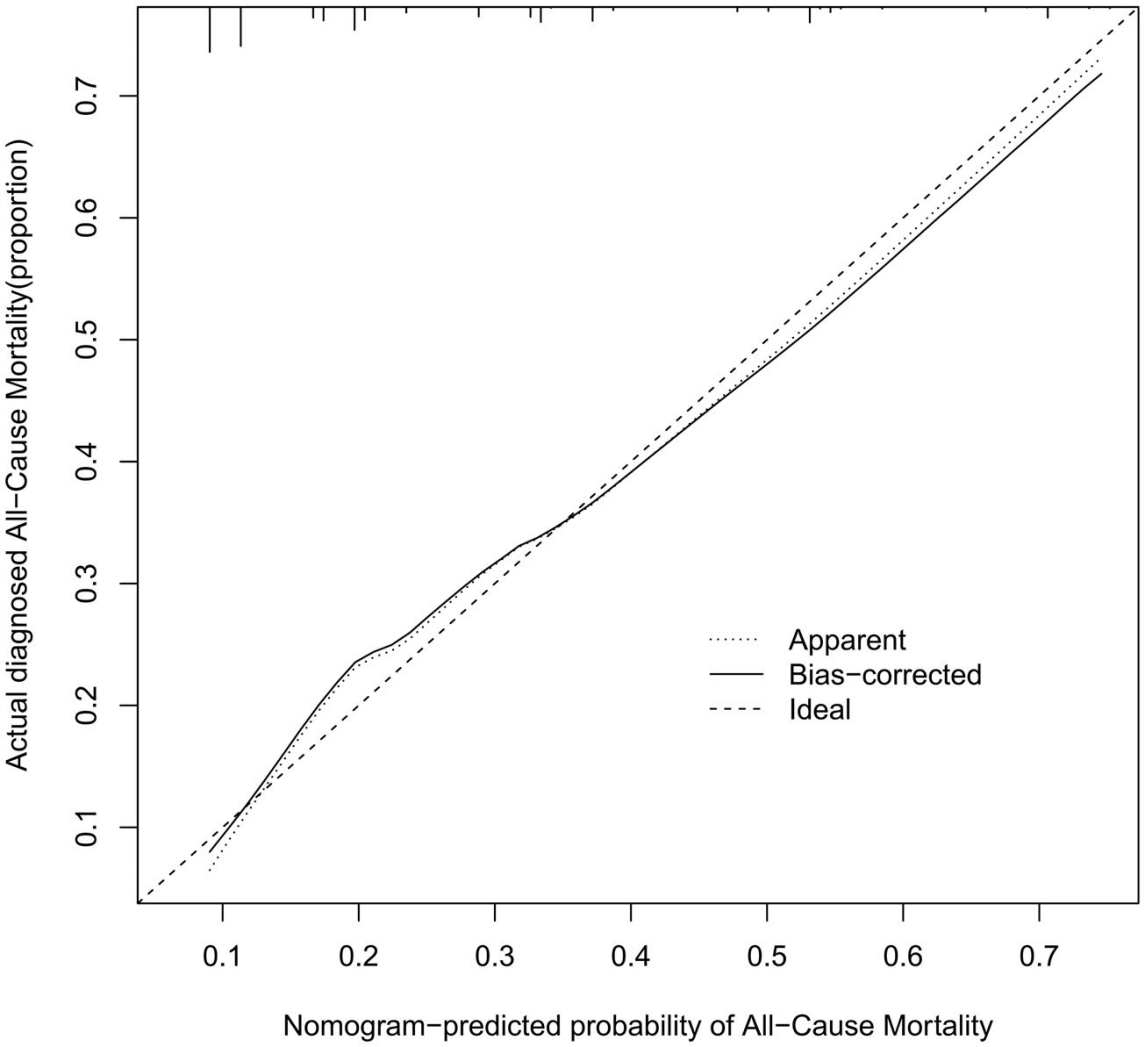
Calibration plots for predicting probability of all-cause mortality. A 45° diagonal line indicates perfect calibration. Calibration plot of training cohort.

**Figure 6.**
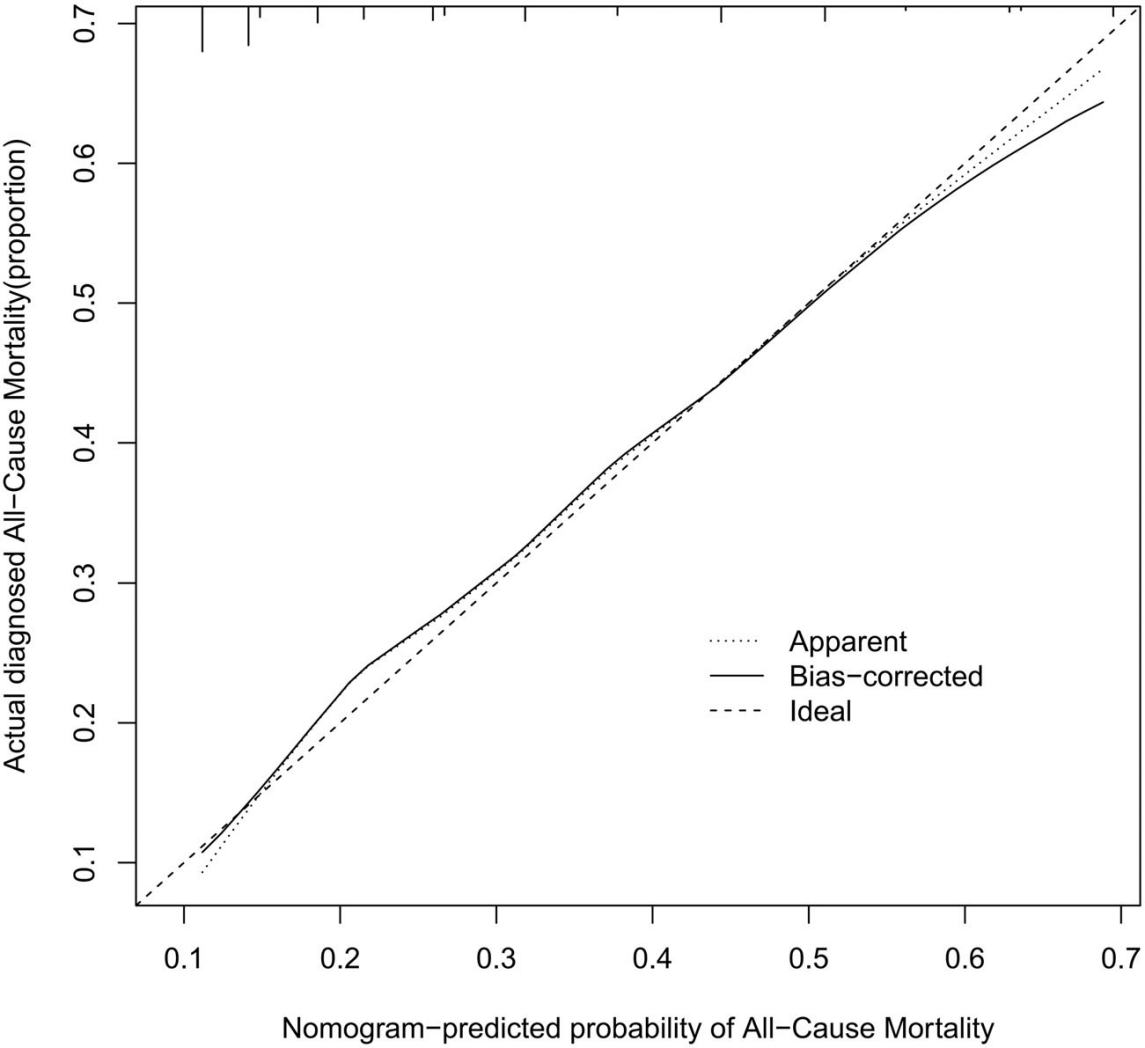
Calibration plots for predicting probability of all-cause mortality. A 45° diagonal line indicates perfect calibration. Calibration plot of validation cohort.

**Figure 7.**
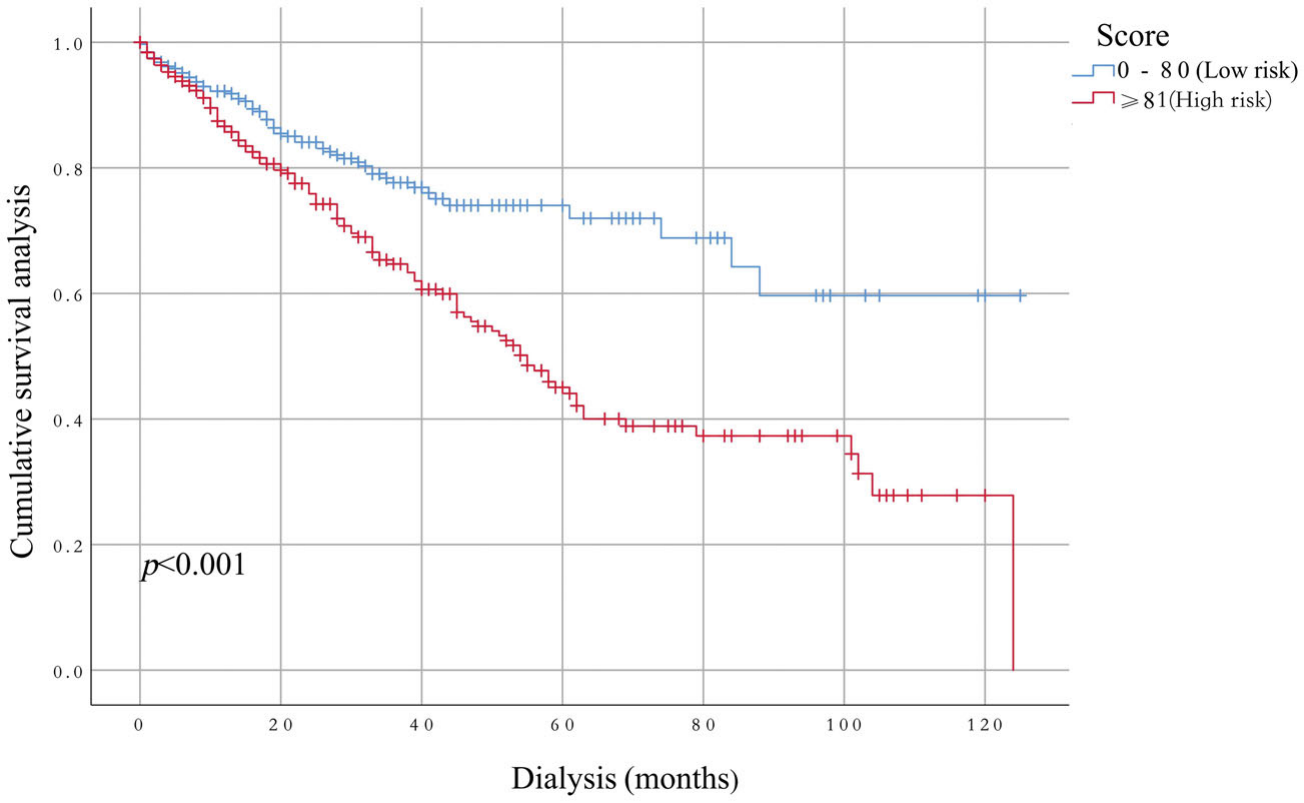
Kaplan–Meier’s survival curves in the training cohort on the basis of the nomogram.

**Figure 8.**
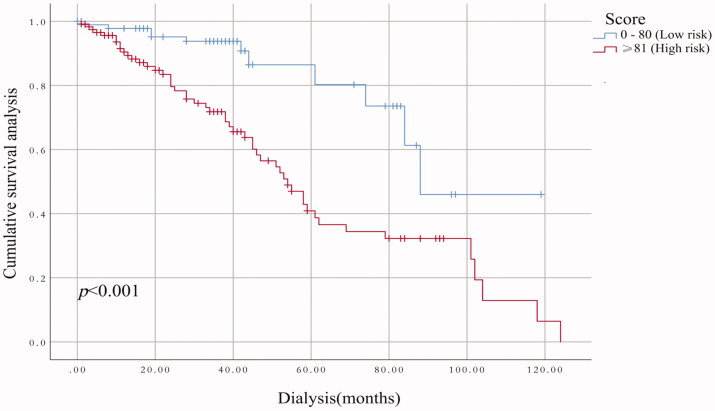
Kaplan–Meier’s survival curves in the validation cohort on the basis of the nomogram.

The performance of the model in the training and validation cohorts was assessed using discrimination and calibration. The model showed good discrimination through ROC curves in two cohorts (Figures 3 and 4). The score revealed good discrimination in the training and validation cohort (AUC 0.779 and 0.758). As given in the calibration plot (Figures 5 and 6), the model appeared to be well-calibrated and a good fit of the predicted probabilities; observed proportions were indicated. Based on these seven predictors, we used a nomogram to develop a score for the prediction of survival probability, the total possible points for the score ranged from 0 to 159 and according to the classification and regression tree model, it was divided into two survival risk levels: low risk (0–80 points) and high risk (≥81 points), and the Kaplan–Meier curves were plotted for these two risk groups (Figures 7 and 8).

### Clinical utility

**Figure 9.**
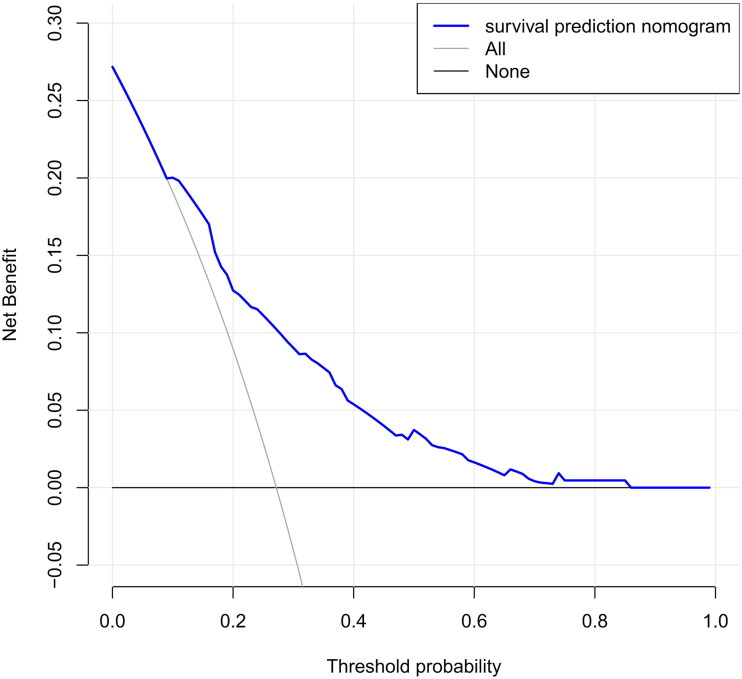
Decision curve analysis for the survival nomogram.

The DCA of the nomograms is presented in Figure 9, the net benefit was calculated by adding the true positives and subtracting the false positives. The gray line represents the assumption that all patients will die, and the horizontal line represents the assumption that no patients will die. The DCA demonstrated that the nomogram added more net benefit compared with the treat-all strategy or treat-none strategy with a threshold probability of 10% or greater.

## Discussion

We developed and validated a easy-to-use nomogram model for all-cause mortality risk among HD patients using seven easily available baseline variables to inform these patients about their future risk up to 10 years, incorporating the result of their clinical features. The prediction nomogram achieved sufficient accuracy and well discrimination which identified that this nomogram can be widely and accurately used for clinical doctors. In addition, the results may be used as guidance for preventive therapy, such as high Hgl and hypoglycemic therapy for patients with a high risk of mortality. However, on the basis of current risk prediction model, comparative studies must be conducted to assess the effectiveness of preventive treatment.

Nomograms have frequently been used in cancer prognosis [11]; however, in recent years, nomograms have been widely used in the prediction of terminal events in various fields and diseases, and have good predictive effect and clinical guidance value. Cheng et al. [12] constructed a nomogram to predict the risk of patients with diabetic kidney disease initiating renal replacement in 3 years. Xia et al. [13] developed and externally validated a nomogram-based model for predicting cardiovascular mortality in incident PD patients, Anker et al. [14] developed a risk-score for 2-year cardiovascular mortality in a Fresenius Medical Care-based HD patients, furthermore, Tang and coworkers [15] developed the nomogram for the estimation of 3-year all-cause mortality using an echocardiography-based risk score in HD patients. Still, to date, a nomogram that can predict 10-year survival is unattainable.

We developed a survival prediction model, which allowed early identification of HD patients at high risk of all-cause mortality. Hence, therapy decisions will be better performed and early interventions will benefit high risk HD patients, and perhaps the most attractive aspect of our nomogram model is its clinical feasibility and simple of use in a wide variety of department of medicine and health. For example, age 80 years who is an HD patient, is nonanemia (Hgl: 120 g/L), nondiabetic, and without CVD, HD was performed using AVF without history of emergency temporary dialysis catheter placement and has a caregiver, will have a total risk score of 54 points, which corresponds to a 1-, 5-, and 10-year probability of survival of 93%, 68%, and 46%, respectively (Figure 2). In contrast, age 50 years who is an HD patient living alone, is diabetic, and with CVD, and has a history of emergency temporary dialysis catheter placement and HD is currently performed using semi-permanent dialysis caregiver, will have a total risk score of 111 points, corresponding to a 1-, 5-, and 10-year probability of survival of 77%, 26%, and 7%, respectively (Figure 2). The easy-to-use nomogram is relatively direct to understand and can be obtained in a short time using a simple form, or by using the online risk scoring calculator, the scores were counted to determine the risk rating of each HD patient at the beginning of dialysis treatment.

In this study, we conducted a multivariable analysis of the included variables, and the results showed that age was an independent risk factor for death in HD patients. Elderly patients are prone to severe complications with poor body resistance and cognitive decline, quality of life decreases and mortality increases after initiation of dialysis, in particular, HD patients who lived alone and did not have caregivers during or after the HD treatment had a higher risk of death. As can be seen from the results of this study, the absence of caregivers was also significantly associated with increased mortality in HD patients; therefore, for elderly patients, especially those without caregivers, nurses should be increased to accompany them, meanwhile, doctors need to evaluate the potential complications more closely during dialysis treatment.

The results also showed that CVD was an independent risk factor for death, previous studies have reported that CVD is the main cause of death in ESRD patients [16], our study found that CVD was the leading cause of death in HD patients, accounting for 37.36% of all deaths, so clinicians need to pay more attention to these patients, such as closely monitoring the fluctuation of blood pressure before and after dialysis. Another important finding was that HD patients with diabetes had a higher risk of death, which may be associated with an increased risk of co-infection in diabetics, and diabetic nephropathy patients with insulin resistance, hyperinsulinemia, and hyperglycemia, thus increasing the risk of CVD and death [17]. This study shows that low Hgl (<90 g/L) is closely related to the mortality of HD patients, anemia is the most common complication in ESRD patients, and improving the anemia status of patients before dialysis can reduce the mortality [18], studies have shown that higher Hgl levels can reduce the risk of left ventricular hypertrophy, increase left ventricular ejection fraction, and improve NYHA classification in ESRD patients [19]. The present study also showed that compared with those who used AVF to perform dialysis treatment, patients received semi-permanent dialysis catheters in early dialysis or patients who had a history of emergency temporary dialysis catheter placement have a higher risk of death. Previous studies have shown that compared with patients with AVF at the initial stage of dialysis, the risk of death in patients who use dialysis catheter is 1.43 times higher [20], the increased risk of death associated with temporary catheter may be caused by unplanned and delayed dialysis treatment, or associated with catheter-related infections. Studies have shown that about 13.3% of patients who were using dialysis catheters have positive blood culture results, while the risk of blood-borne infection in catheter patients is three times higher than that in AVF patients [21]. Other studies have shown that these potential infection risks and inflammatory states were associated with increased cardiovascular risk [22,23], the consistent evidence is that white blood cells and Hs-CRP in the dead patients were significantly higher than those in the survival patients in this study, suggesting a possible link with more severe inflammatory state caused by catheters; therefore, effective evaluation of vascular conditions in HD patients before dialysis, preparation for establishment of dialysis pathway in advance, and increasing the proportion of AVF in the initial treatment may effectively reduce the risk of death.

Although previous studies have done some good predictions of mortality in HD patients, for example, the vascular calcification score (AUC = 0.72), Anker’s model (c-index = 0.72–0.74), and Shastri’s model (c-index = 0.75) performed effective in predicting CVD mortality for HD patients [14,24,25], compared with these three prediction models, our current nomogram performed favorable discrimination as reported by an AUC of 0.779 for the training cohort and 0.758 for the validation cohort. Furthermore, our model demonstrated good calibration based on Kaplan–Meier’s survival curves for both training and validation cohort, we advocate the use of our nomogram for estimating near-term, intermediate-term, and long-term survival in HD patients. Doubtlessly, to ensure the stability of our model, the need for repetition and further validation of our results in other well-defined populations is necessary.

However, there are some limitations in this study. First of all, this study is a single-center study with a small sample size, which may increase the possibility of type II errors, and we developed a nomogram for predicting survival, but external validation was absent. At the same time, only those variables with univariate analysis results of *p*< .05 were selected for the Cox analysis, which to some extent lost the related risk factors affecting death, so a larger sample size study was needed to confirm the findings of this study. Last, although the robustness of our nomogram was examined extensively with internally validation using bootstrap testing, the universality was uncertain for other HD patients, it needs to be externally assessed in wider HD populations.

## Conclusions

This study developed a easy-to-use nomogram with good accuracy for predicting 1-, 5-, and 10-year survivals in HD patients. The simple and reliable score was designed to identify HD who were at high risk of death; therefore, this nomogram suggested treatment of anemia, disordered blood glucose levels, and CVD may be the key points to reduce all-cause mortality risk for HD patients; furthermore, it is also possible to improve the survival rate by reducing the use of semi-permanent dialysis catheter and avoiding temporary dialysis catheter implantation in case of emergency; the planned establishment of AVF prior to dialysis is advocated, in addition, having a caregiver for dialysis patient is also an important way to improve the survival.

## Data Availability

The data used in this study are available from the corresponding author upon request.

## Abbreviations

ACEi: angiotensin-converting enzyme inhibitor
Alb: albumin
ARB: angiotensin receptor blocker
AVF: arteriovenous fistula
BMI: Body mass index
BUN: blood urea nitrogen
Ca: calcium
CCB: calcium channel blocker
Cr: creatinine
CVD: Cardiovascular disease
HB: Hemoglobin
HD: hemodialysis
Hgl: hemoglobin
Hs-CRP: high-sensitivity C-reactive protein
K: potassium
LDL: low-density lipoprotein cholesterol
P: phosphorus
PTH: parathyroid hormone
RKF: residual kidney function
TC: total cholesterol
TG: total triglycerides
UA: serum uric acid
